# Supplementary material for: WordCommentsAnalyzer: A windows software tool for qualitative research
Source: F1000Res. 2018 Sep 4;7:536. Originally published 2018 May 3. [Version 2] doi: 10.12688/f1000research.14819.2 (PMC6137411; doi:10.12688/f1000research.14819.2)
Supplement: Supplementary file 3 [file f1000research-7-17614-s0002.tgz › b2a79496-4ada-47c4-9682-a99ce867836d.zip!/Eight Iranian Health Journals/Archives of Rehabilitation_45.docx]

© Archives of Rehabilitation. This work is licensed under CC BY-NC 4.0 (https://creativecommons.org/licenses/by-nc/4.0/).

Volume & Issue: دوره 9 - شماره 2

Title: بررسی تأثیر ماساژ نقاط فشاری بر کیفیت خواب سالمندان

http://rehabilitationj.uswr.ac.ir/article-1-247-fa.html

هدف: سالمندان اغلب از اختلال خواب رنج می برند و طب فشاری سنتی چینی یکی از تکنیکهای غیر تهاجمی است که از طریق ماساژ نقاط فشاری سبب تعادل انرژی در بدن و ارتقاء راحتی و سلامتی می‌شود. هدف این مطالعه بررسی تأثیر ماساژ نقاط فشاری بر کیفیت خواب سالمندان دارای اختلال خواب است. روش بررسی: در این مطالعه نیمه تجربی که به‌صورت کارآزمایی بالینی یک سوکور انجام گرفت، از پرسشنامه شاخص کیفیت خواب پیتزبرگ (PSQI) جهت غربال و انتخاب نمونه‌های دچار مشکل خواب استفاده شد. نمونه‌ها به روش نمونه گیری مبتنی بر هدف انتخاب و به روش تصادفی تعادلی در سه گروه نقاط حقیقی طب فشاری، گروه نقاط غیر حقیقی و گروه کنترل قرار گرفتند. درهرسه گروه تعداد نمونه‌ها 30 نفر و به نسبت مساوی از هر دو جنس بودند. ماساژ نقاط مورد نظر در گروه نقاط حقیقی طب فشاری و گروه نقاط غیر حقیقی به‌طور یکسان 15 دقیقه، سه روز در هفته و به مدت چهار هفته و در مورد گروه کنترل تنها ارتباط کلامی صورت گرفت. در پایان مداخله 77 نفر از نمونه‌ها پرسشنامه را تکمیل نمودند. برای تجزیه و تحلیل داده‌ها از آزمونهای تحلیل واریانس، تحلیل کوواریانس، تحلیل واریانس با اندازه‌گیری‌های مکرر وکروسکال والیس استفاده گردید. یافته ها: بر اساس نتایج تحلیل داده ها، بعد از مداخله اختلاف عمده‌ای در نمرات مقیاس‌های فرعی کیفیت خواب ذهنی (P=0/025)، مدت‌زمان خواب واقعی (P=0/012)، کفایت خواب (P=0/022)، اختلالات خواب (P=0/025)، عملکرد روزانه (P=0/033)، خواب نهفته (P<0/001) و میانگین نمره کل شاخص کیفیت خواب پیتز برگ (P<0/001) بین سه گروه نقاط حقیقی طب فشاری، گروه نقاط غیر حقیقی و گروه کنترل وجود داشت. در ادامه آزمون تعقیبی اختلاف عمده‌ای را از نظر مقیاسهای فرعی و نمره کل شاخص کیفیت خواب پیتزبرگ بین گروه نقاط حقیقی و گروه کنترل نشان داد (P<0/05). درحالی‌که بین گروه نقاط حقیقی و گروه نقاط غیر حقیقی تنها از نظر خواب نهفته (P=0/006) و نمره کل شاخص کیفیت خواب پیتز برگ (P=0/021) اختلاف معنی دار وجود داشت. داده‌های حاصل از برگه ثبت روزانه خواب بهبود معنی دار کیفیت خواب و کاهش تعداد بیداری شبانه را طی مداخله نشان داد (P<0/001). نتیجه‌گیری: این مطالعه تأثیر طب فشاری بر بهبود کیفیت خواب سالمندان را مورد تأیید قرار داده و طب فشاری سنتی چینی را به عنوان یک روش درمان غیر دارویی برای سالمندان دچار اختلال خواب پیشنهاد می‌نماید.

Title: تأثیر کاشت حلزون بر گیجی، افسردگی، اضطراب و سلامت روان بزرگسالان کم‌شنوای پس از زبان‌آموزی

http://rehabilitationj.uswr.ac.ir/article-1-248-fa.html

هدف: کم‌شنوایی می‌تواند بر سلامت جسمی و روانی- اجتماعی افراد تأثیر گذاشته و موجب ضعف خودباوری، زودرنجی، انزواطلبی، نا امیدی، افسردگی و اضطراب آنها شود. کاشت حلزون به عنوان یکی از وسایل کمک شنوایی منجر به بهبود شرایط روانشناختی افراد کم شنوا می‌گردد. مطالعه حاضر با هدف بررسی تأثیر کاشت حلزون بر گیجی، افسردگی، اضطراب و سلامت عمومی بزرگسالان کم‌شنوایی پس از زبان‌آموزی صورت گرفته است. روش بررسی: در این مطالعه مقطعی – مقایسه‌ای که به صورت مورد – شاهدی انجام شد، به روش ساده و در دسترس 24 فرد کم شنوا که تحت عمل کاشت حلزون قرار گرفته بودند و 25 فرد کم‌شنوا که کاندید دریافت کاشت حلزون بودند با رعایت همتاسازی از بیمارستانهای شهر تهران انتخاب و گیجی، افسردگی، اضطراب و سلامت عمومی آنها با استفاده از پرسشنامه‌های هندیکپ گیجی (DHI)، افسردگی بک ویرایش دوم (BDI-II)، اضطراب بک (BAI) و سلامت عمومی (GHQ) مورد مقایسه قرار گرفت. داده‌ها به کمک روش آماری تحلیل واریانس چندمتغیره (MANOVA) تجزیه و تحلیل گردید. یافته‌ها: میانگین نمرات افسردگی و اضطراب مؤید کاهش این شاخصه‌های روانشناختی در گروه کاشت حلزون شده است که این کاهش در افسردگی معنادار است (P=0/001). در مورد سلامت عمومی، تفاوت معناداری بین دو گروه مشاهده نشد (P=0/415). میانگین نمرات گیجی نشان دهنده افزایش این اختلال در گروه کاشت حلزون شده است (P=0/004). نتیجه‌گیری: به نظر می‌رسد کاشت حلزون در کاهش افسردگی و اضطراب و بهبود وضعیت روانشناختی افراد کم شنوا مؤثر باشد، اگرچه این تأثیر در مورد اضطراب و سلامت عمومی خیلی چشمگیر نیست. همچنین کاشت حلزون منجر به گیجی بیشتر می‌شود.

Title: بررسی تعامل بین توانایی پردازش توالی‌های شناختی و درک نحوی در بیماران فارسی‌زبان مبتلا به زبان‌پریشی بروکا

http://rehabilitationj.uswr.ac.ir/article-1-250-fa.html

هدف: فرآیند‌های فیزیولوژیکی - عصبی مشترک یا به هم مرتبطی برای پردازش ساخت انتزاعی غیر زبانی مانند توالی‌های شناختی و بعضی از جنبه‌های ساخت نحوی زبان لازم است. از آنجا که تاکنون در ایران تحقیقی با هدف بررسی رابطه بین توانایی پردازش توالی‌های شناختی و درک ساخت‌های نحوی دارای ترتیب سازه‌ای نامتعارف در بیماران فارسی زبان مبتلا به زبان‌پریشی بروکا انجام نشده است، این پژوهش با همین هدف انجام شد. روش بررسی: در یک مطالعه شبه تجربی با طرح پیش آزمون- پس آزمون، 10 بیمار مرد (6 نفر) و زن (4 نفر) فارسی زبان که مطابق آزمون زبان‌پریشی نیلی پور، مبتلا به زبان پریشی بروکا بودند، مورد بررسی قرار گرفتند. نمونه‌های مورد بررسی از میان بیماران مبتلا به زبان‌پریشی مراجعه کننده به کلینیک‌های گفتاردرمانی دولتی شهر تهران به‌صورت ساده و مبتنی بر هدف انتخاب شدند. درک نحوی بیماران قبل و بعد از یک دوره آموزشی 10 هفته‌ای در زمینه توالی‌های شناختی (هر هفته یک جلسه) با استفاده از آزمونی که بر اساس آزمون درک نحوی در زبان‌پریشی برای زبان فارسی بومی شده بود، مورد سنجش قرار گرفت و توانایی پردازش توالی‌های شناختی بر اساس آزمون للکوف ارزیابی شد. درصد پاسخ‌های درست بیماران در آزمون نحوی و آزمون للکوف محاسبه و نتایج به‌دست‌آمده به وسیله آزمون‌ تی زوجی مورد تجزیه و تحلیل قرار گرفت. یافته‌ها: بین توانایی درک ساخت‌های نحوی که دارای ترتیب سازه‌ای نامتعارف هستند و توانایی پردازش توالی‌های شناختی بیماران ارتباط مستقیم وجود داشت (ضریب همبستگی=0/50). پس از دوره آموزش، توانایی درک ساخت‌هایی که دارای ترتیب سازه‌ای نامتعارف در زبان فارسی هستند، به طور معناداری افزایش یافت (P<0/05)، ولی توانایی درک ساخت‌های دارای ترتیب سازه‌ای متعارف تغییر معناداری نکرد (P>0/05). توانایی پردازش توالی‌های شناختی بیماران در طول دوره آموزش افزایش یافته و اختلاف پیش آزمون و پس‌آزمون معنادار بود (P<0/05). نتیجه‌گیری: تقویت توانایی پردازش توالی‌های پیچیده سبب بهبود توانایی درک ساخت‌های دارای ترتیب سازه‌ای نامتعارف در بیماران مبتلا به زبان‌پریشی بروکا می‌شود، اما بر توانایی آنها در درک ساخت‌های دارای ترتیب سازه‌ای متعارف تأثیری ندارد.

Title: تجزیه و تحلیل کلام بیماران اسکیزوفرنیک از جنبه اصل همیاری گرایس

http://rehabilitationj.uswr.ac.ir/article-1-251-fa.html

هدف: هدف این پژوهش بررسی یکی از حیطه‌های تجزیه و تحلیل کلام بیماران اسکیزوفرنیک یعنی اصل همیاری می‌باشد. روش بررسی: در این پژوهش مقایسه‌ای که از نوع مورد- شاهدی است، با نمونه‌گیری ساده و در دسترس، دو گروه 22 نفره از بیماران مبتلا به اسکیزوفرنیا و افراد سالم (با تعداد 17 مرد و 5 زن) انتخاب و مصاحبه‌ای با استفاده از بخش گفتار خودبه‌خودی آزمون زبان پریشی فارسی با آنها صورت گرفت. پاسخ هر سئوال با توجه به این که فرد کدام قاعده همیاری (کیفیت، کمیت، شیوه و رابطه) را بیشتر نقض کرده است، ارزیابی و به عنوان خطا در آن پاسخ در نظر گرفته شد. با درصد گیری خطاها،‌ تجزیه و تحلیل داده‌ها با استفاده از آزمون‌های تحلیل واریانس، تی مستقل، کروسکال والیس و من ویتنی انجام شد. یافته‌ها: بین دو گروه در رعایت کلی اصول همیاری گرایس (P<0/001) و اصلهای کمیت (P<0/001)، رابطه (P<0/001) و شیوه (P=0/02) اختلاف معنی‌داری وجود داشت، ولی در اصل کیفیت دو گروه تفاوت معنی‌داری نداشتند (P=0/07). عدم رعایت کلی اصول گرایس در بیماران با مدت ابتلا به بیماری، تحصیلات، سن و جنس آنها ارتباط آماری معنی‌دار نداشت (P<0/05). نتیجه گیری: بیماران اسکیزوفرنیک اصل همیاری گرایس را رعایت نمی‌کنند و به علت نقض این اصل در گفتگوهای دو طرفه دچار عدم انسجام می‌‌باشند.

Title: تأثیر درمان یکپارچگی حسی بر مهارت‌های حرکتی درشت و ظریف کودکان 5 تا 7 ساله با نشانگان داون

http://rehabilitationj.uswr.ac.ir/article-1-253-fa.html

هدف: کودکان مبتلا به نشانگان داون دچار کژکاری در پردازش و یکپارچگی حسی هستند که می‌تواند موجب تأخیر در رشد مهارت‌های حرکتی درشت و ظریف آنها شود. هدف پژوهش حاضر بررسی تأثیر درمان یکپارچگی حسی بر مهارت‌های حرکتی درشت و ظریف کودکان 5 تا 7 ساله مبتلا به نشانگان داون بود. روش بررسی: شرکت کنندگان در این مطالعه تجربی شامل 60 کودک 7-5 ساله با نشانگان داون بودند که به‌صورت تصادفی از چند مرکز توانبخشی شهر تهران انتخاب و با انتصاب تصادفی ساده در گروه‌های مداخله و کنترل قرارگرفتند. مقیاس‌های رشد حرکتی پی بادی به‌عنوان پیش‌آزمون و پس آزمون برای ارزیابی مهارتهای حرکتی درشت و ظریف به کار گرفته شد. داده‌های حاصل با استفاده از آزمون‌های آماری از جمله آزمون لون، آزمون تی مستقل و تحلیل کوواریانس مورد تجزیه و تحلیل قرار گرفت. یافته‌ها: بین نمرات پیش‌آزمون و پس آزمون حرکات درشت شرکت‌کنندگان در گروه‌های مداخله و کنترل از لحاظ آماری تفاوت معناداری وجود داشت (P<0/001). در حرکات ظریف این تفاوت فقط در گروه مداخله معنادار بود (P<0/001) و در گروه مقایسه تفاوت معناداری مشاهده نشد (P=0/013). همچنین بین میانگین اختلاف نمرات پیش‌آزمون و پس‌آزمون شرکت‌کنندگان در گروه‌های مداخله و کنترل در حرکات درشت (P<0/001) و ظریف (P<0/001) تفاوت معناداری وجود داشت. نتیجه‌گیری: یافته‌های حاصل نشان می‌دهد که درمان یکپارچگی حسی موجب بهبود مهارت‌های حرکتی درشت و ظریف کودکان 5 تا 7 ساله با نشانگان داون می‌شود. پیشنهاد می‌شود که این رویکرد درمانی با التفات به نیازهای این کودکان در برنامه‌های توانبخشی آنها گنجانده شود.

Title: تأثیر بهبود تقارن در تحمل وزن بر ثبات پاسچر در بیماران همی‌پارزی

http://rehabilitationj.uswr.ac.ir/article-1-254-fa.html

هدف: اختلال در کنترل پاسچر به عنوان مهمترین عامل زمین‌خوردن در افراد همی‌پارزی به دنبال سکته مغزی شناخته شده است. یکی از مشخصات بارز عدم تعادل پاسچرال در این بیماران، عدم تقارن در تحمل وزن روی اندام تحتانی است. هدف این مطالعه بررسی تأثیر بهبود تقارن از طریق مداخلات درمانی بر ثبات پاسچر در بیماران همی‌پارزی بود. روش بررسی: در این مطالعه شبه تجربی که از نوع قبل و بعد می‌باشد، 27 بیمار همی‌پارزی به روش در دسترس و ساده از بین بیماران همی‌پارزی مراجعه‌کننده به درمانگاه اعصاب بیمارستان حضرت رسول انتخاب شدند. جهت ارزیابی انحراف پاسچرال از بیماران خواسته شد با چشمان باز و بسته روی صفحات نیرو بایستند. ارزیابی‌ها شامل اندازه‌گیری شاخص عدم تقارن، ریشه دوم میانگین (RMS) نوسان مرکز فشار و RMS سرعت نوسان مرکز فشار در صفحات فرونتال و ساژیتال بود. در مرحله بعد در حالی‌ که یک کفی 10 میلی‌متری زیر پای غیردرگیر بیمار قرار داده شده بود، ارزیابی‌های اولیه تکرار ‌شد. جهت تجزیه و تحلیل داده‌ها از آزمون آماری تحلیل واریانس برای اندازه‌گیریهای مکرر استفاده شد. یافته‌ها: تأثیر خالص کفی بر شاخص عدم تقارن معنی‌دار بود (P<0/0001). کفی و حالت بینایی تعاملی با یکدیگر نداشته و تأثیر خالص کفی بر شاخصه‌های ثبات پاسچر معنی‌دار نبود (مقدار احتمال برای RMS نوسان مرکز فشار در صفحات فرونتال و ساژیتال به ترتیب P=0/186 و P=0/245 و برای RMS سرعت نوسان مرکز فشار در صفحات فرونتال و ساژیتال به ترتیب P=0/259 و P=0/342 بدست آمد). نتیجه‌گیری: قراردادن کفی با مایل نمودن مرکز گرانش به سمت خط وسط بدن، باعث بهبود تقارن می‌شود. با توجه به این که بهبود تقارن باعث کاهش بی‌ثباتی پاسچرال نگردید، تحمل وزن نامتقارن نمی‌تواند عامل اصلی بی ثباتی پاسچرال باشد. لذا درصورت تأیید این مطلب در مطالعات دیگر و بدون محدودیت‌های این تحقیق، بهبود تقارن نباید هدف اصلی برنامه‌های توانبخشی باشد که به دنبال بهبود تعادل ایستاده هستند.

Title: مقایسه تأثیر خستگی بر پاسخ الکترومیوگرافی عضله تراپزیوس در اغتشاش خلفی-قدامی در افراد سالم و مبتلا به گردن درد مزمن

http://rehabilitationj.uswr.ac.ir/article-1-256-fa.html

هدف: عضلات افراد مبتلا به گردن درد، خستگی پذیری سریعتری نسبت به افراد سالم نشان داده و خستگی این عضلات نوسان تنه را در حالت ایستاده افزایش می‌دهد. از آنجا که درباره پاسخ این عضلات در شرایط دینامیک مانند اغتشاش خارجی، اطلاعات کمی وجود دارد، لذا این تحقیق با هدف مقایسه تأثیر خستگی عضلانی بر زمان پاسخ عضله تراپزیوس فوقانی در اغتشاش خلفی- قدامی، در افراد سالم و مبتلایان به گردن درد انجام پذیرفت. روش بررسی: در این مطالعه شبه تجربی و مداخله‌ای، با روش نمونه‌گیری ساده و در دسترس، 16 بیمار مبتلا به گردن ‌درد مزمن و 16 فرد سالم با توجه به همتاسازی دو گروه و درنظر داشتن معیارهای ورود و خروج انتخاب شدند. جمع‌آوری داده‌ها با استفاده از پرسشنامه، قبل و پس از مداخله انجام و ابزارهای اصلی شامل نیروسنج، شتاب سنج و دستگاه الکترومیوگرافیک سطحی بود. برای ایجاد خستگی، از وزنه‌ای معادل 30 درصد حداکثر قدرت ایزومتریک و برای ایجاد اغتشاش از وزنه‌ای برابر با ده درصد وزن بدن استفاده شد. در تجزیه و تحلیل داده‌ها از آمار توصیفی، آزمون تی زوجی، تی مستقل و تحلیل واریانس با اندازه‌گیریهای مکرر استفاده گردید.  یافته‌ها: اختلاف معنی‌داری بین زمان پاسخ عضله تراپزیوس در اغتشاش خلفی- قدامی بین دو گروه، قبل (P=0/006) و بعد از خستگی (P=0/026) وجود داشت. در افراد سالم پس از خستگی زمان پاسخ طولانی‌تر و در افراد مبتلا زمان پاسخ کوتاهتر ‌شد. بین زمان پاسخ عضله‌ تراپزیوس در اغتشاش خلفی – قدامی قبل و پس از خستگی در گروه سالم (P=0/04) و گروه بیماران (P<0/001) تفاوت معنی‌‌دار بود. نتیجه‌گیری: درد می‌تواند سبب تغییر در زمان پاسخ عضلانی، کاهش فعالیت عضلات عمقی و تغییر الگوی فعالیت عضلانی شود و خستگی به دلیل تشدید این شرایط و افزایش خطر می‌تواند سبب کاهش زمان پاسخ عضلات سطحی در افراد مبتلا به گردن درد مزمن و فیکس شدن سیستم جهت ایجاد ثبات شود، که این موضوع خود خطر آسیب را بالا می‌برد.

Title: مقایسه دانش زبانی بین دانش‌آموزان دوزبانه‌ مبتلا به لکنت و سالم

http://rehabilitationj.uswr.ac.ir/article-1-255-fa.html

هدف: گزارش‌های زیادی درباره ارتباط بین لکنت و رشد زبان مطرح شده است. برخی از تحقیقات نشان داده‌اند که دوزبانگی می‌تواند عاملی تأثیرگذار در ابتلا به لکنت باشد. هدف اصلی این مطالعه، مقایسه‌ دانش زبانی کردی و فارسی بین دانش‌آموزان دوزبانه‌ مبتلا به لکنت و سالم شهر جوانرود است. روش بررسی: مطالعه‌ مقطعی - مقایسه‌ای حاضر از نوع مورد- شاهدی است. 31 دانش‌آموز دوزبانه‌ مبتلا به لکنت با روش تمام‌شمار به عنوان گروه مورد و 31 دانش‌آموز سالم با روش همسان‌سازی جفتی به عنوان گروه شاهد از دانش‌آموزان پایه‌های چهارم و پنجم مقطع ابتدایی شهر جوانرود انتخاب شدند. دانش زبانی کردی و فارسی دو گروه، از طریق مقایسه‌ شاخص‌های کیفیت گفتار (روانی گفتار، پیچیدگی گفتار، غنای واژگانی) در نمونه‌ گفتار توصیفی آزمودنی‌ها بررسی و داده‌های حاصل با استفاده از آزمون‌ تی مستقل و آزمون یو مان - ویتنی مورد تجزیه و تحلیل قرار گرفت. یافته‌ها: در دو زبان کردی و فارسی شدت ناروانی گفتار در گروه مبتلا به لکنت بیشتر و پیچیدگی گفتار، روانی گفتار و غنای واژگانی گروه مبتلا به لکنت کمتر از گروه سالم و در تمامی مشخصه‌های شاخص‌های گفتاری فوق اختلاف دو گروه معنادار بود (P<0/05). نتیجه‌گیری: مطابق نتایج حاصل از این تحقیق دانش زبانی افراد دوزبانه مبتلا به لکنت در هر دو زبان اول و دوم کمتر از گروه سالم می‌باشد. این یافته مؤید این نکته است که سطح مهارت زبانی احتمالاً به عنوان یکی از عوامل تأثیرگذار بر لکنت مطرح است.

Title: مقایسه تولید واکه‌های ساده فارسی در کودکان کاشت حلزون‌شده براساس سن کاشت

http://rehabilitationj.uswr.ac.ir/article-1-257-fa.html

هدف: سن کاشت حلزون یکی از عوامل مؤثر در میزان موفقیت به‌کارگیری این پروتز جهت کسب مهارتهای گفتاری و زبانی کودکان کم شنوا است. از آنجا که تولید صحیح واکه‌ها به‌عنوان یک توانمندی ارتباطی در گفتار شاخص مهمی است، لذا هدف این پژوهش، مقایسه تولید واکه‌های ساده فارسی بین کودکان کم شنوایی که قبل و بعد از سن دو سالگی مورد عمل کاشت حلزون قرار گرفته‌اند و بررسی تفاوتهای بین آنها می‌باشد. روش بررسی: این تحقیق، به صورت مقطعی – مقایسه‌ای، بر روی 70 کودک که قبل از 2 سالگی و 70 کودک که بعد از 2 سالگی مورد عمل کاشت حلزون قرار گرفته و به‌صورت ساده از نمونه‌های در دسترس در بیمارستانهای امیراعلم و حضرت رسول (ص) تهران و با توجه به معیارهایی مثل داشتن صحت جسمی و ذهنی کامل، تک زبانه بودن، گذراندن 1±9 ماه توانبخشی، نداشتن والدین کم شنوا و عدم سابقه بیماریهای خاص انتخاب شده بودند، انجام شد. همچنین 238 کودک طبیعی به‌عنوان گروه معیار به‌صورت تصادفی ساده از مهدکودکهای شهر تهران انتخاب شدند. شش واکه ساده فارسی /ای، اِ، اَ، آ، اُ، او/ براساس نمره میانگین فورمنت فرکانسهای اول و دوم واکه‌ها بین سه گروه مقایسه گردید. جهت تجزیه و تحلیل فورمنت فرکانسهای واکه‌ای از ویرایش 2/1 نرم‌افزار اکوستیکی SFSwin و جهت تحلیل داده‌ها از آزمون آماری تی مستقل استفاده شد. یافته‌ها: میانگین فورمنت فرکانسهای واکه‌ای F2/u/ (P=0/006)، F2/o/ (P=0/012)، F2/a/ (P=0/012) / F2/æ (P=0/039)، F2/e/ (P=0/006)، F1/e/ (011/0P=) و F2/i/ (046/0P=) در دو گروه دارای اختلاف معناداری بود، ولی اختلاف نمره میانگین فورمنت فرکانسهای واکه‌ای F1/u/، F1/o/، F2/a/،‌ F2/æ/ و F1/i/ بین دو گروه معنادار نبود (P<0/05). فورمنت فرکانسهای واکه‌ای هیچ یک از شش واکه ساده فارسی بین گروه زیر دو سال کاشت‌شده و گروه طبیعی تفاوت معناداری نداشت (P<0/05). نتیجه‌گیری: با توجه به وجود اختلاف معنادار در تولید واکه‌ها بین دو گروه کودکان کاشت حلزون شده و عدم اختلاف معنادار بین کودکانی که زیر 2 سالگی کاشت حلزون شده‌اند با کودکان طبیعی، می‌توان نتیجه گرفت که انجام کاشت حلزون در سنین زیر 2 سال در کودکان کم‌شنوا، از بسیاری مشکلات احتمالی در مهارتهای گفتاری و زبانی و خراب‌گویی‌های واکه‌ای آنها جلوگیری خواهد کرد.

Title: مدیریت حوزه سلامت در بلایای طبیعی با تأکید بر توانبخشی

http://rehabilitationj.uswr.ac.ir/article-1-258-fa.html

بلایا اتفاقات معمول و روزمره نیستند. آنها از هر نوعی که باشند، طبیعی یا انسان ساخت، زندگی و فعالیت‌های هر جامعه‌ای را مختل کرده و کلیه منابع فردی و اجتماعی را تخریب می‌نمایند. در شرایط پس از بلیه، بعد از شوک اولیه، بازگردانیدن زندگی و معاش مردم به شرایط طبیعی از مهمترین دغدغه‌های جوامع و ملت‌هاست. به‌طور سنتی این مرحله تحت عنوان توانبخشی و بهبودی شناخته شده است. بازتوانی در حوادث به معنی بازگردانیدن جامعه به شرایط قبل از بلیه است. در حقیقت فرایندی است که ممکنست سالیان درازی طول کشیده و تمام جنبه‌های فیزیکی، اجتماعی و اقتصادی جامعه را در بر بگیرد. از آنجا که بلایا حیات انسان را تهدید می‌کنند، بنابراین آماده بودن تنها راه دستیابی به نتایج بهتر است. برنامه‌ریزی صحیح قبل از وقوع بلیه و استفاده هماهنگ از منابع می‌تواند منجر به پاسخی مؤثر و مناسب به بحران شود. در این برنامه آمادگی، باید ضمن تأکید روی مشارکت مدیران محلی بازسازی ساختارهای مدیریتی نیز صورت گیرد.

Title: گزارش یک مورد نقص مادرزادی قسمت فوقانی ران

http://rehabilitationj.uswr.ac.ir/article-1-259-fa.html

نقص مادرزادی قسمت فوقانی ران اختلال نادری است که شیوع آن 1 نفر در 50 هزار تا 200 هزار تولد زنده گزارش شده است. در این بیماری سر ران و غالباً حفره استابولوم درگیر می‌شود که می‌تواند بصورت یک طرفه و یا دوطرفه باشد. مشکلات ناشی از این نقص مادرزادی می‌تواند به صورت های مختلف نظیر تفاوت درطول اندامها، بی ثباتی مفصل ران و زانو، انقباض عضلات ران و زانو، سطح نامساوی زانوها، کوتاهی قد و دیگر مشکلات همراه بروز نماید. معمولاً کودکان مبتلا و خانواده های آنها در طی درمان با تصمیم گیری های متفاوتی اعم از درمانهای جراحی و غیرجراحی مواجه می‌شوند که حتی در بین متخصصین نیز این درمانها بحث‌انگیز  است. در مقاله حاضر، پسربچه 3 ساله ای مبتلا به این نوع نقص مادرزادی با اختلال تکامل شدید در سر استخوانهای ران وحفره استابولوم، کوتاهی استخوان ران بصورت دوطرفه و تأخیر در مراحل رشد حرکتی معرفی می‌شود که با توصیه پزشک به منظور دریافت خدمات کاردرمانی و توان بخشی به دانشکده توانبخشی مراجعه کرده بود.
